# Serological Evidence of Exposure to Globally Relevant Zoonotic Parasites in the Estonian Population

**DOI:** 10.1371/journal.pone.0164142

**Published:** 2016-10-10

**Authors:** Brian Lassen, Marilin Janson, Arvo Viltrop, Kädi Neare, Pirje Hütt, Irina Golovljova, Lea Tummeleht, Pikka Jokelainen

**Affiliations:** 1 Department of Basic Veterinary Sciences and Population Medicine, Institute of Veterinary Medicine and Animal Science, Estonian University of Life Sciences, Tartu, Estonia; 2 Department of Veterinary Disease Biology, University of Copenhagen, Frederiksberg C, Denmark; 3 Institute of Biomedicine and Translational Medicine, Chair of Medical Microbiology and Virology, Faculty of Medicine, University of Tartu, Tartu, Estonia; 4 Department of Virology, National Institute for Health Development, Tallinn, Estonia; 5 Faculty of Veterinary Medicine, University of Helsinki, Helsinki, Finland; Universidade de Aveiro, PORTUGAL

## Abstract

We investigated Estonian population and its selected subgroups for serological evidence of exposure to *Ascaris lumbricoides*, *Echinococcus* spp., *Taenia solium*, *Toxocara canis*, *Toxoplasma gondii*, and *Trichinella spiralis*. Serum samples from 999 adults representing general population, 248 children aged 14–18, 158 veterinarians, 375 animal caretakers, and 144 hunters were tested for specific immunoglobulin G antibodies against the selected parasites using commercial enzyme immunoassays (ELISA). Sera yielding positive or twice grey zone *Echinococcus* spp, *T*. *solium*, *T*. *canis*, and *T*. *spiralis* results were subjected to western blot (WB) analysis. In the general population, based on the ELISA results, the *A*. *lumbricoides* seroprevalence was 12.7%, *Echinococcus* spp. seroprevalence was 3.3%, *T*. *solium* seroprevalence was 0.7%, *T*. *canis* seroprevalence was 12.1%, *T*. *gondii* seroprevalence was 55.8%, and *T*. *spiralis* seroprevalence was 3.1%. *Ascaris lumbricoides* seroprevalences were higher in children and in animal caretakers than in the general population, and *T*. *canis* seroprevalence was higher in animal caretakers than in the general population. Compared with the general population, *Echinococcus* spp. seroprevalence was higher in children. By contrast, *T*. *gondii* seroprevalence was higher in animal caretakers, and lower in children, than in the general population. In the general population, the WB-confirmed *Echinococcus* spp. seroprevalence was 0.5%, *T*. *solium* cysticercosis seroprevalence was 0.0%, *Toxocara* spp. seroprevalence was 14.5%, and *Trichinella* spp. seroprevalence was 2.7%. WB-confirmed *Toxocara* spp. seroprevalence was higher in animal caretakers than in the general population. We found serological evidence of exposure to zoonotic parasites in all tested groups. This calls for higher awareness of zoonotic parasitic infections in Estonia.

## Introduction

Comprehensive studies on exposure to zoonotic parasites are needed [1, 2]. Zoonoses present a challenge to public health and wealth, and some groups, such as children and immunocompromised persons, are more vulnerable [3, 4]. Zoonotic infections can also be an occupational risk for groups including veterinarians, animal caretakers, and hunters [5, 6, 7, 8, 9].

Recent research confirms that several zoonotic parasites are common and endemic in Estonia, which is located in north-eastern Europe [10, 11, 12, 13, 14, 15, 16, 17]. We designed a cross-sectional serological study to investigate the exposure to *Ascaris* spp., *Echinococcus* spp., *Taenia solium*, *Toxocara canis*, *Toxoplasma gondii*, and *Trichinella spiralis* in the Estonian population and its four subgroups: children aged 14–18, animal caretakers, hunters, and veterinarians.

The selected parasites are ranked high among zoonotic parasites that were evaluated for their global relevance as foodborne pathogens [1, 2]: *T*. *solium* as the 1st, *E*. *granulosus* 2nd, *E*. *multilocularis* 3rd, *T*. *gondii* 4th, *T*. *spiralis* 7th, *Ascaris* spp. 9th, *Trichinella* spp. 16th and *Toxocara* spp. 20th [1].

The highest reported incidence of ascariosis was 2702 per 100000 inhabitants in 1955 [18]. Between 2000 and 2012, the median incidence was 24.1 per 100000 inhabitants [19, 20, 21, 22].

*Echinococcus* spp. are endemic in the Baltic countries, and the incidence of human cases has increased [14]. This is in conflict with the statement that the risk of acquiring echinococcosis in Estonia would be negligible [23]. Until 2014, official reports mention 13 cases of human echinococcosis, four of which were classified as imported [14].

There are no available reports of human infections with *Toxocara* spp. from Estonia.

The highest reported incidence of *T*. *solium* infections was 14.8 per 100000 inhabitants in 1959 [18]. Official Estonian public health information mentions two human *T*. *solium* infections from 2000–2001 [24].

The local *T*. *gondii* seroprevalence has been high: in the town of Tartu, 61.8% in 1991–1993 [25] and 54.9% in 1999–2001 [26]. Seropositivity indicates chronic infection with the parasite. Since 1999, 78 cases of toxoplasmosis have been reported in Estonia [20, 21, 22], including three cases of congenital toxoplasmosis: two from 2002 and one from 2003 (1.54 and 0.77 per 10000 births, respectively).

The highest reported incidence of trichinellosis was 2.8 per 100000 inhabitants in 1993 [18]. Since 1999, 13 human trichinellosis cases have been reported in Estonia [20, 21, 22, 23].

In this nationwide study, we aimed to estimate the seroprevalences of the selected zoonotic parasites, and to evaluate the differences in seroprevalence between the general population and the subgroups. Our hypothesis was that people in Estonia would have serological evidence of exposure to all of the parasites, and that in certain subgroups, the seroprevalences would be higher compared to general population.

## Material and Methods

### Ethics Statement

The study was approved by the Research Ethics Committee of the University of Tartu (nr. 216/T-15, 227/M-5 and 235/M-26).

The general population samples were obtained from a biobank (http://www.geenivaramu.ee/en) and the children samples were obtained from a sample collection (National Institute for Health Development, http://tai.ee/en/). There had been no formal signed informed consent of a parent or guardian of the children, but written information had been given and it had been emphasized that the participation was voluntary. The veterinarians, animal caretakers, and hunters gave written informed consent before the blood samples were taken by nurses. The sera were stored and analysed coded.

Those veterinarians, animal caretakers, and hunters who had provided contact information were informed of their serology results and given a short description of what seropositivity means. In addition, they were provided the contact information for designated research group members, to whom further questions could be addressed. Those with medical questions were guided to consult their own family physician.

### Setting

Estonia is located in the north-eastern Europe and has a population of 1.3 million inhabitants [27]. Approximately 1000 veterinary practitioners are licenced to work in the country [28]. The number of persons working as animal caretakers is unknown. There are over 15000 hunters [29].

### Samples

The general population samples (n = 999), from individuals 18 years and older, were obtained as a random sample stratified by county and gender from the serum bank of the Estonian Genome Center. The samples had been collected in 2004–2011.

Sera from apparently healthy children aged 14–18 years (n = 248) were obtained from a serum bank that had been collected in 2003. The samples originated from different parts of the country.

Veterinarians (n = 158) were sampled at a local veterinary conference in October 2012.

Animal caretakers (n = 375) included persons involved with dairy cattle (n = 193), beef cattle (n = 51), pigs (n = 68), and sheep and goats (n = 63). From those involved with dairy cattle, blood samples were collected in March–May 2013, whereas those involved with beef cattle were sampled in January 2014. Animal caretakers involved with pigs were sampled in September–November 2013, and those involved with sheep and goats were sampled in October–November 2013. Those involved with pigs were sampled during farm visits, whereas the collection of the other samples was arranged at local professional meetings and events.

Hunters (n = 144) were sampled during a national meeting of hunters in July 2013.

General population samples and children samples were stored frozen at -20°C and thawed prior to the analyses. The samples from veterinarians, animal caretakers, and hunters were allowed to clot and then centrifuged, at the sampling site. The sera were separated within 24 hours. The sera were stored at +4°C for up to two days, during which the first analyses were performed, and then frozen at -20°C until thawed prior to further analyses.

### Serological analyses

The serum samples were tested using NovaLisa IgG enzyme immunoassays (ELISA) (NovaTec Immunodiagnostica GmbH, Dietzenbach, Germany) for the presence of immunoglobulin G (IgG) antibodies against *A*. *lumbricoides* (specificity (Sp) 95%), sensitivity (Se) >95%), *Echinococcus* spp. (Sp >95%, Se >95%), *T*. *solium* (Sp >95%, Se 93.8%), *T*. *gondii* (Sp 98.2%, Se 96.6%), *T*. *canis* (Sp >95%, Se >95%), and *T*. *spiralis* (Sp 94.8%, Se >95%), according to the manufacturer’s instructions. The controls provided in the kits were used in each analysis. They included standards A, B, C, and D for the *T*. *gondii* ELISA, and positive control, cut-off control, and negative control for the other ELISAs.

The samples that tested positive with ELISA were considered seropositive. The samples that yielded a grey zone result were retested, and the second test result was considered the final ELISA result.

Samples that had tested positive or yielded a grey zone result twice with the *Echinococcus* spp., *T*. *solium*, *T*. *canis* and *T*. *spiralis* ELISA were further tested using ECHINOCOCCUS Western Blot IgG, CYSTICERCOSIS Western Blot IgG, TOXOCARA Western Blot IgG, and TRICHINELLA Western Blot IgG (LDBIO DIAGNOSTICS, Lyon, France), respectively. The positive controls provided in the kits were used in each analysis. The results were interpreted following the manufacturer’s instructions.

The samples that tested positive with the western blot (WB) were considered confirmed seropositives. It is noteworthy that the *T*. *solium* ELISA is intended for detecting antibodies against *T*. *solium* antigens, both taeniasis and cysticercosis, while the corresponding WB is based on antigens from crude larval extract and intended for detecting cysticercosis caused by the larval stages of the parasite only.

The crude seroprevalences were calculated using the number of seropositives (ELISA result) as the numerator. The WB-confirmed seroprevalences were calculated using the number of confirmed seropositives (ELISA and WB in series) as the numerator. The true seroprevalences (Rogan-Gladen) were calculated using the number of seropositives (ELISA result) and taken into account the Sp and Se, with EpiTools [30]. If the Sp or Se was given as “>95%”, the calculation was done using 95%. The denominator was the number of samples tested in each group.

### Exclusion of samples

The database was searched for double entries: one sample from general population and one sample from animal caretakers (persons involved with beef cattle) were excluded.

### Statistical analyses

The statistical analyses were performed using the free software OpenEpi [31]. Confidence intervals (95% CI) were calculated using Mid-P exact test. Two-by-two tables were used to evaluate differences in crude seroprevalences between the groups. Bonferroni adjustment was used to reduce the likelihood of type 1 error, and differences with two-tailed *p*-values < 0.01 (Mid-P exact test) were considered statistically significant. When comparing a single result of ours to another published result, a difference with two-tailed *p*-value < 0.05 (Mid-P exact test) was considered statistically significant.

## Results

### Ascaris lumbricoides

The *A*. *lumbricoides* seroprevalence was 12.7% in the general population. All groups showed evidence of exposure to *A*. *lumbricoides* (Table 1). The seroprevalence was significantly higher in children and in animal caretakers than in the general population (*p* < 0.001).

**Table 1:** *Ascaris lumbricoides* ELISA results including those that tested positive (POS) and those that tested positive or yielded a grey zone result twice (POS+GREY) in the general population, children, veterinarians, animal caretakers, and hunters in Estonia.

	General	Children	Veterinarians	Animal	Hunters
	population			caretakers	
	(n = 999)	(n = 248)	(n = 158)	(n = 375)	(n = 144)
ELISA (POS)					
n positive	127	78	14	81	20
Prevalence (%)	12.7	31.5 **	8.9	21.6 **	13.9
95% CI	10.8–14.9	25.9–37.4	5.1–14.1	17.7–26.0	8.9–20.3
ELISA (POS+GREY)					
n positive	212	101	17	136	33
%	21.2	40.7 **	10.8 *	36.3 **	22.9
95% CI	18.8–23.8	34.7–46.9	6.6–16.3	31.5–41.2	16.6–30.3

Comparison with the general population:

* *p* ≤ 0.01

** *p* ≤ 0.001

CI = confidence interval

The true *A*. *lumbricoides* seroprevalence was 8.6% (95% CI 6.3–10.9) in the general population, 29.4% (95% CI 23.0–35.8) in children, 4.3% (95% CI -0.6–9.2) in veterinarians, 18.4% (95% CI 13.8–23.1) in animal caretakers, and 9.9% (95% CI 3.6–16.2) in hunters.

### *Echinococcus* spp.

The *Echinococcus* spp. seroprevalence was 3.3% and the WB-confirmed *Echinococcus* spp. seroprevalence was 0.5% in the general population. All groups showed evidence of exposure to *Echinococcus* spp. (Table 2). The seroprevalence was higher in children (*p* < 0.001) than in the general population. WB-confirmed seropositives were detected in the general population and animal careretakers group.

**Table 2:** *Echinococcus* spp. ELISA and western blot (WB) results including those that tested positive (POS) and those that tested positive or yielded a grey zone result twice (POS+GREY) in the general population, children, veterinarians, animal caretakers, and hunters in Estonia.

	General	Children	Veterinarians	Animal	Hunters
	population			caretakers	
	(n = 999)	(n = 248)	(n = 158)	(n = 375)	(n = 144)
ELISA (POS)					
n positive	33	30	3	4	1
%	3.3	12.1 **	2.0	1.1	0.7
95% CI	2.3–4.6	8.5–16.6	0.5–5.1	0.3–2.6	0.0–3.4
ELISA (POS+GREY)					
n positive	94	13	4	34	2
%	9.4	34.3	2.5 *	9.1	1.4 **
95% CI	7.7–11.3	28.6–40.4	0.8–6.0	6.5–12.3	0.2–4.5
WB (POS)					
n postive	2	0	0	0	0
%	0.2	0.0	0.0	0.0	0.0
95% CI	0.0–0.7	0.0–1.2	0.0–1.9	0.0–0.8	0.0–2.1
WB (POS+GREY)					
n positive	5	0	0	1	0
%	0.5	0.0	0.0	0.3	0.0
95% CI	0.2–1.1	0.0–1.2	0.0–1.9	0.0–1.3	0.0–2.1

Comparison with the general population:

* *p* ≤ 0.01

** *p* ≤ 0.001

CI = confidence interval

The true *Echinococcus* spp. seroprevalence was <0% (95% CI -3.1-(-0.7)) in the general population, 7.9% (95% CI 3.4–12.4) in children, <0% (95% CI -5.8-(-1.1)) in veterinarians, <0% (95% CI -5.5-(-3.2)) in animal caretakers, and <0% (95% CI -6.3-(-3.3)) in hunters.

### Taenia solium

The *T*. *solium* seroprevalence was 0.7% and the WB-confirmed *T*. *solium* cysticercosis seroprevalence was 0.0% in the general population. Seropositive individuals were detected in all groups except the children (Table 3).

**Table 3:** *Taenia solium* ELISA and cysticercosis western blot (WB) results including those that tested positive (POS) and those that tested positive or yielded a grey zone result twice (POS+GREY) in the general population, children, veterinarians, animal caretakers, and hunters in Estonia.

	General	Children	Veterinarians	Animal	Hunters
	population			caretakers	
	(n = 999)	(n = 248)	(n = 158)	(n = 375)	(n = 144)
ELISA (POS)					
n positive	7	0	1	3	2
%	0.7	0.0	0.6	0.8	1.4
95% CI	0.3–1.4	0.0–1.2	0.0–3.1	0.2–2.2	0.2–4.5
ELISA (POS+GREY)					
n positive	15	1	1	5	5
%	1.5	0.4	0.6	1.3	3.5
95% CI	0.9–2.4	0.0–2.0	0.0–3.1	0.5–2.9	1.3–7.5
WB (POS)					
n positive	0	0	0	0	0
%	0.0	0.0	0.0	0.0	0.0
95% CI	0.0–0.3	0.0–1.2	0.0–1.9	0.0–0.8	0.0–2.1
WB (POS+GREY)					
n positive	0	0	0	0	0
%	0.0	0.0	0.0	0.0	0.0
95% CI	0.0–0.3	0.0–1.2	0.0–1.9	0.0–0.8	0.0–2.1

CI = confidence interval

The true *T*. *solium* seroprevalence was <0% (95% CI -6.8-(-5.6)) in the general population, <0% (95% CI -7.0-(-7.0)) in children, <0% (95% CI -7.7-(-4.9)) in veterinarians, <0% (95% CI -7.1-(-5.1)) in animal caretakers, and <0% (95% CI -7.6-(-3.3)) in hunters.

### Toxocara canis

The *T*. *canis* seroprevalence was 12.1% and the WB-confirmed *Toxocara* spp. seroprevalence was 14.5% in the general population. All groups showed evidence of exposure to *T*. *canis* (Table 4). The seroprevalence was higher in animal caretakers (*p* < 0.001) than in the general population. The WB-confirmed *Toxocara* spp. seroprevalence was also higher in animal caretakers (*p* < 0.001) than in the general population.

**Table 4:** *Toxocara canis* ELISA and *Toxocara* spp. western blot (WB) results including those that tested positive (POS) and those that tested positive or yielded a grey zone result twice (POS+GREY) in the general population, children, veterinarians, animal caretakers, and hunters in Estonia.

	General	Children	Veterinarians	Animal	Hunters
	population			caretakers	
	(n = 999)	(n = 248)	(n = 158)	(n = 375)	(n = 144)
ELISA (POS)					
n positive	121	42	12	97	21
%	12.1	16.9	7.6	25.9 **	14.6
95% CI	10.2–14.3	12.7–22.0	4.2–12.6	21.6–30.5	9.5–21.1
ELISA (POS+GREY)					
n positive	145	51	15	110	25
%	14.5	20.6	9.5	29.3 **	17.4
95% CI	12.4–16.8	15.9–25.9	5.6–14.8	24.9–34.1	11.8–24.2
WB (POS)					
n positive	121	41	12	95	21
%	12.1	16.9	7.6	25.3 **	14.6
95% CI	10.2–14.3	12.7–22.0	4.2–12.6	21.1–29.9	9.5–21.1
WB (POS+GREY)					
n positive	145	50	15	108	25
%	14.5	20.2	9.5	28.8 **	17.4
95% CI	12.4–16.8	15.5–25.5	5.6–14.8	24.4–33.5	11.8–24.2

Comparison with the general population:

** *p* ≤ 0.001

CI = confidence interval

The true *T*. *canis* seroprevalence was 7.9% (95% CI 5.7–10.2) in the general population, 13.3% (95% CI 8.1–18.4) in children, 2.9% (95% CI -1.7–7.5) in veterinarians, 23.2% (95% CI 18.3–28.1) in animal caretakers, and 10.6% (95% CI 4.2–17.1) in hunters.

### Toxoplasma gondii

The *T*. *gondii* seroprevalence was 55.8% in the general population. All groups showed evidence of exposure to *T*. *gondii* (Table 5). The seroprevalence was higher in animal caretakers (*p* < 0.01), and lower in children (*p* < 0.001), than in the general population.

**Table 5:** *Toxoplasma gondii* ELISA results including those that tested positive (POS) and those that tested positive or yielded a grey zone result twice (POS+GREY) in the general population, children, veterinarians, animal caretakers, and hunters in Estonia.

	General	Children	Veterinarians	Animal	Hunters
	population			caretakers	
	(n = 999)	(n = 248)	(n = 158)	(n = 375)	(n = 144)
ELISA (POS)					
n positive	557	93	73	279	94
%	55.8	37.5 **	46.2	74.4 **	65.3
95% CI	52.7–58.8	31.6–43.7	38.5–54.0	69.8–78.6	57.2–72.7
ELISA (POS+GREY)					
n positive	564	94	73	281	94
%	56.0	37.9 **	46.2	74.7 **	65.3
95% CI	53.0–59.1	32.0–44.0	38.5–54.0	70.1–78.9	57.2–72.7

Comparison with the general population:

** *p* ≤ 0.001

CI = confidence interval

The true *T*. *gondii* seroprevalence was 55.2% (95% CI 52.0–58.5) in the general population, 36.0% (95% CI 29.6–42.3) in children, 45.2% (95% CI 37.0–53.4) in veterinarians, 74.9% (95% CI 70.2–79.6) in animal caretakers, and 65.3% (95% CI 57.1–73.5) in hunters.

### Trichinella spiralis

The *T*. *spiralis* seroprevalence was 3.1% and the WB-confirmed *Trichinella* spp. seroprevalence was 2.7% in the general population. All groups showed evidence of exposure to *T*. *spiralis* (Table 6).

**Table 6:** *Trichinella spiralis* ELISA and *Trichinella* spp. western blot (WB) results including those that tested positive (POS) and those that tested positive or yielded a grey zone result twice (POS+GREY) in the general population, children, veterinarians, animal caretakers, and hunters in Estonia.

	General	Children	Veterinarians	Animal	Hunters
	population			caretakers	
	(n = 999)	(n = 248)	(n = 158)	(n = 375)	(n = 144)
ELISA (POS)					
n positive	31	8	5	13	8
%	3.1	3.2	3.2	3.5	4.9
95% CI	2.2–4.3	1.5–6.0	1.7–6.9	1.9–5.7	2.2–9.4
ELISA (POS+GREY)					
n positive	44	15	5	18	8
%	4.4	6.0	3.2	4.8	5.6
95% CI	3.3–5.8	3.6–9.6	1.7–6.9	3.0–7.3	2.6–10.3
WB (POS)					
n positive	18	2	4	9	4
%	1.8	0.8	2.5	2.4	2.8
95% CI	1.1–2.8	0.1–2.6	0.8–6.0	1.2–4.4	0.9–6.6
WB (POS+GREY)					
n positive	27	2	4	11	5
%	2.7	0.8	2.5	2.9	3.5
95% CI	1.8–3.9	0.1–2.6	0.8–6.0	1.6–5.0	1.3–7.5

CI = confidence interval

The true *T*. *spiralis* seroprevalence was <0% (95% CI -3.3-(-0.9)) in the general population, <0% (95% CI -4.4–0.5) in children, <0% (95% CI -5.1–0.1) in veterinarians, <0% (95% CI -3.8–0.4) in animal caretakers, and 0.6% (95% CI -3.5–4.8) in hunters.

## Discussion

We detected evidence of exposure to all of the zoonotic parasites tested. These zoonotic parasites present a threat to human health and life quality, animal health and welfare, food safety, the economy, and the environment [1, 32, 33]. The results of this study call for higher awareness of zoonotic parasitic infections in Estonia.

The *A*. *lumbricoides* seroprevalence in general population was lower than an estimate from the Netherlands (33.0%, *p* < 0.001) [34]. The *A*. *lumbricoides* seroprevalence estimate in children in Estonia was higher than estimates from Poland (15.0%, *p* < 0.001) [35] and from the Netherlands (7.2%, *p* < 0.001) [36]. In our study, the seroprevalence was higher in the children than in adults. This is in contrast to an increase in seroprevalence with age that was observed in the Netherlands [34]. One possible explanation could be that exposure to this parasite would have increased recently in Estonia, but there are no direct data to support that. The higher seroprevalence in animal caretakers might be due to contact with *Ascaris* eggs in the agricultural environment.

The *Echinococcus* spp. seroprevalence estimate in general population was higher than that observed in Austria (0.0%, *p* < 0.001) [37] and Greece (1.1%, *p* < 0.01) [38], but similar to that observed in Poland (3.2%) [39] and Spain (3.4%) [40]. The seroprevalence in veterinarians was similar to that in veterinary surgeons in Turkey (2.2%) [41]. The *Echinococcus* spp. seroprevalence in hunters was lower than both *E*. *granulosus* seroprevalence (10.7%, *p* < 0.001) and *E*. *multilocularis* seroprevalence (5.4%, *p* < 0.05) in hunters in Austria [7], while none of the seropositives in either of the studies tested positive with WB. The data available on *Echinococcus* spp. infections in animal hosts originates mainly from research projects, but *E*. *multilocularis*, *E*. *canadensis* (G8 and G10) of *E*. *granulosus* (G1) have been diagnosed in animal hosts in Estonia recently [14].

We found no comparable data on *T*. *solium* seroprevalence, while the parasite is considered relevant in Europe [42, 43]. Cysticercosis has been reported from Estonian pigs [44].

The *T*. *canis* seroprevalence in general population was higher than the estimates from Austria (6.3%, *p* < 0.001) [45], Sweden (7.1%, *p* < 0.01) [45], Denmark (2.6%, *p* < 0.001) [46], and the Netherlands (8.3%, *p* < 0.001) [34], but similar to that from Poland (13.0%) [39] and that from the Slovak Republic 20 years ago (13.7%) [47]. The WB-confirmed *Toxocara* spp. *s*eroprevalence was higher than a similarly confirmed estimate from Denmark (2.4%, *p* < 0.001) [46] and higher than people with epilepsy in Italy when tested with a comparable method (6.5%, *p* < 0.01) [48]. The *T*. *canis* seroprevalence and the WB-confirmed *Toxocara* spp. seroprevalence in hunters were similar to those in hunters in Austria (16.8%) [7]–in both studies, all seropositive hunters tested positive also with WB. The WB-confirmed *Toxocara* spp. seroprevalence in children was higher than a similarly confirmed estimate from children from Poland (4.2%, *p* < 0.001) [49]. *Toxocara* spp. are endemic in animal hosts in Estonia [50, 51, 52], and *Toxocara* spp. eggs have been found shed into the urban environment [51, 52].

The *T*. *gondii* seroprevalences were worryingly high when compared with recent results from other European countries, where the seroprevalence has decreased [53, 54]. The burden caused by *T*. *gondii* infections is high [1, 2, 32] and the parasite merits attention. *Toxoplasma gondii* seroprevalence typically increases with age, indicating acquired infections. The high *T*. *gondii* seroprevalence in children in Estonia suggests that the infection pressure is substantial, while different age distribution might partly explain some of the differences noted between other groups. Contact with contaminated environment on farms [55] may partly explain the higher seroprevalence in farm workers. High *T*. *gondii* seroprevalence in domestic cats in Estonia [15] indicates that the environment has been contaminated with oocysts, which is supported by results from wild and domestic animals [12, 16].

The *T*. *spiralis* seroprevalence in the general population was similar to an estimate in forest workers in Poland (6.0%) [39], and the WB-confirmed *Trichinella* spp. seroprevalence was similar to an estimate in hunting communities in Greenland (3.3%) [56]. *Trichinella* spp. merit higher awareness as relevant zoonotic parasites in Estonia, in animal hosts particularly in the sylvatic cycle [17].

The sample sizes were adequate for estimating and comparing the seroprevalences. The general population samples were a good representation of the Estonian population. The children group only included samples from youngsters aged over 14 years; thus the seroprevalences in younger children remain unknown. The convenience samples from children, veterinarians, animal caretakers, and hunters may be limited by geographical representativeness; those interested in research activities and further professional education may be overrepresented.

Serology is an indirect detection method. For detecting chronic *T*. *gondii* infections, serology is widely used, whereas for other parasites, serology results should be interpreted with more caution. Detecting antibodies provides evidence of exposure and can indicate infection [57] and has been used in follow-up of patients. For example, patients with alveolar echinococcosis appear to maintain ELISA-seropositivity despite the intensity of the WB bands may fade during the follow-up, while a curative resection results in seronegativity in some patients in a few years [58].

We chose to investigate the presence of IgG antibodies because they are commonly long-lasting and suitable for epidemiological studies. However, investigating only one class of antibodies is a limitation of the study.

The assays used are based on purified antigens, but some potential cross-reactions are listed by the manufacturer. Of the 499 *T*. *canis* positive samples, 130 (26.1%) tested positive for antibodies against *A*. *lumbricoides* and ten (2.0%) tested positive for antibodies against *Echinococcus* spp. with the corresponding ELISA assays. A majority (99.1%) of *T*. *canis* results tested positive with the corresponding WB. One sample that had tested positive for antibodies against *T*. *canis* and for antibodies against *Echinococcus* spp. tested positive for both with the corresponding WB assays.

Overall, it appeared to be a good decision to include also samples that yielded a grey zone ELISA result twice to be tested with WB. Several samples yielded a grey zone result twice with ELISA but tested positive with WB (Tables 2, 4 and 6).

The methods were evaluated to be suitable for an epidemiological study, although communicating an individual result required explaining the main limitations of the methods used. Informing the veterinarians, animal caretakers, and hunters of their results was evaluated to be an ethically reasoned choice.

These results provide baseline data, which can inform public health decision makers and suggest where further research and prevention efforts should be targeted. It is obvious that the zoonotic parasites circulating in Estonia reach also humans, but the locally relevant risk factors for encountering the parasites are currently largely unknown.

## Conclusions

People living in Estonia had evidence of having been exposed to several zoonotic parasites, which calls for evaluation of need of prevention strategies and higher awareness. Antibodies against zoonotic parasites appeared to be formed already in childhood, indicating considerable infection pressure. The results suggest that zoonotic parasitic infections are underdiagnosed or underreported in Estonia.
